# Biological Nano-Agrochemicals for Crop Production as an Emerging Way to Address Heat and Associated Stresses

**DOI:** 10.3390/nano14151253

**Published:** 2024-07-26

**Authors:** József Prokisch, Aya Ferroudj, Safa Labidi, Hassan El-Ramady, Eric C. Brevik

**Affiliations:** 1 Nanofood Laboratory, Department of Animal Husbandry, Institute of Animal Science, Biotechnology and Nature Conservation, Faculty of Agricultural and Food Sciences and Environmental Management, University of Debrecen, 138 Böszörményi Street, 4032 Debrecen, Hungary; jprokisch@agr.unideb.hu (J.P.); ferroudj.aya@agr.unideb.hu (A.F.); safe@mailbox.unideb.hu (S.L.); hassan.elramady@agr.kfs.edu.eg (H.E.-R.); 2 Soil and Water Department, Faculty of Agriculture, Kafrelsheikh University, Kafr El-Sheikh 33516, Egypt; 3 College of Agricultural, Life, and Physical Sciences, Southern Illinois University, Carbondale, IL 62901, USA

**Keywords:** climate change, nanofertilizers, nanopesticides, drought stress, salinity stress, nano-management, nanotoxicity

## Abstract

Climate change is a global problem facing all aspects of the agricultural sector. Heat stress due to increasing atmospheric temperature is one of the most common climate change impacts on agriculture. Heat stress has direct effects on crop production, along with indirect effects through associated problems such as drought, salinity, and pathogenic stresses. Approaches reported to be effective to mitigate heat stress include nano-management. Nano-agrochemicals such as nanofertilizers and nanopesticides are emerging approaches that have shown promise against heat stress, particularly biogenic nano-sources. Nanomaterials are favorable for crop production due to their low toxicity and eco-friendly action. This review focuses on the different stresses associated with heat stress and their impacts on crop production. Nano-management of crops under heat stress, including the application of biogenic nanofertilizers and nanopesticides, are discussed. The potential and limitations of these biogenic nano-agrochemicals are reviewed. Potential nanotoxicity problems need more investigation at the local, national, and global levels, as well as additional studies into biogenic nano-agrochemicals and their effects on soil, plant, and microbial properties and processes.

## 1. Introduction

Global climate change represents a serious threat to crop production [1]. Climate change can subject crops to stresses including heat, flooding, salinity, drought, and soil nutrient deficiencies [2]. These stresses can reduce crop productivity by up to 50%, depending on the type of stress [3]. Stresses may occur individually [4] or in combination [2,5,6] that simultaneously or sequentially impact crop yield and the overall health of an agroecosystem [7]. How crops balance their growth and respond to adverse environments or stresses have been reported in several studies [8], which include abiotic stresses [9] such as drought [10], salinity [11], flooding [12], heat [13], heavy metals [14], and ozone [15], as well as biotic stresses [16].

Crop production requires considerable management to alleviate stresses. This often includes agrochemicals such as fertilizers and pesticides. The application of nano-enabled agrochemicals in the agricultural sector has gained considerable attention due to the lack of sustainability of many modern agricultural practices [17]. Reports on the use of nano-agrochemicals to counter biotic and abiotic stresses have included the documentation of smart nano-agrochemicals [18], nanotoxicity on non-target aquatic species [19], influence on the plant-beneficial microbiome [17], enhancing stress tolerance [20], nanopesticide formulations [21], nano-priming [22], drought stress [23], and environmental nano-preservation [24]. Among nano-agrochemical forms, biological nano-formulations are considered the most desirable approach under stressful conditions [20,23,25,26,27,28]. The biological nanomaterials have many promising benefits for the sustainable production of crops due to their high efficiency and low toxicity [20] in alleviating plant abiotic and biotic stresses (through nanopesticides) and improving the delivery of targeted nutrients by nanofertilizers [27].

Heat stress is one of the biggest crop production challenges related to global climate change. Heat stress is not just a challenge on its own but is also associated with other stresses that may link to drought [29,30,31], salinity [32,33,34,35], and biotic stress [36]. The main impact of heat stress is in the production of reactive oxygen species (ROS) that cause oxidative damage to plant cell organelles and their functions [37]. Irreversible injuries can reduce the productivity of crops, causing yield loss and a reduction in food quality, even under short exposure to heat stress [37].

Therefore, this review is an attempt to emphasize the synergistic role of biological nano-agrochemicals in the mitigation of heat stress and other associated stresses during crop production. The promising mitigating effects of biogenic nanofertilizers and nanopesticides on heat stress will be highlighted. Nano-management of heat stress and suggested strategies will be reported as well.

## 2. Nano-Agrochemicals and Crop Production

**Figure 1:**
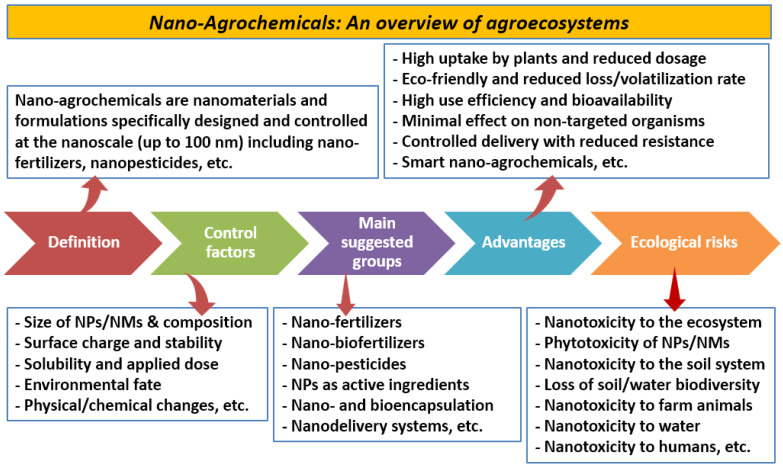
An overview of nano-agrochemicals, including definition, main groups, factors controlling the release of these compounds, benefits, and ecological risks. Abbreviations: NPs—nanoparticles and NMs—nanomaterials.

Nano-agrochemicals can be defined as nanomaterials and/or formulations that are designed and controlled at the nanoscale for agronomic purposes (Figure 1). These compounds have great potential to revolutionize farming practices by promoting the efficiency of agronomic practices using properties of these NMs, such as controlled nutrient release, high use efficiency, high surface area, and reduced ecological risks [17]. Many benefits can be listed for the nano-agrochemicals, such as their eco-friendly nature, reduced loss/volatilization rate, high uptake by plants, reduced applied dosage, high use efficiency and bioavailability, controlled delivery with reduced resistance, and minimal effect on non-targeted organisms. The advantages of nano-agrochemicals have been confirmed for many different crops to enhance their productivity under stressful or/and non-stressful conditions, as reported in the following sections of this review. On the other hand, several ecological risks could occur due to the application of nano-agrochemicals. These risks could result in nanotoxicity to the agroecosystem, including phytotoxicity and nanotoxicity to the soil system, loss of biodiversity in both soil and water, and toxicity to farm animals and humans.

## 3. Stresses Associated with Heat Stress

**Figure 2:**
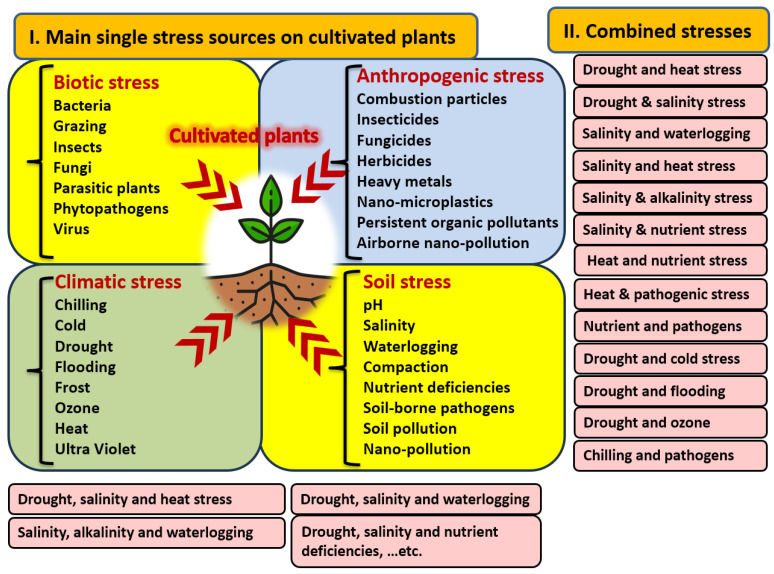
The main sources of stress on cultivated plants in single and combined forms. Heat stress can be found in association with many other stresses, such as drought, salinity, flooding, and nutrient deficiencies. Sources: [7,69,73].

Crops face many stresses that can be classified into four groups: (1) soil, (2) anthropogenic, (3) climate, and (4) biotic stresses (Figure 2). The first three are often referred to as abiotic stresses. Specific stresses related to soil, including abiotic stresses such as pH [38], salinity [39], alkalinity [40], waterlogging [41], compaction [42,43], nutrient deficiencies [44,45], soil pollution [46], and soil nano-pollution [47], as well as biotic stresses such as soil-borne plant pathogens [48] and parasites [49], have been investigated. Anthropogenic pollutants that can stress plants include particles from combustion [50], pesticides [51], heavy metals [52], nano-microplastics [53], persistent organic pollutants [54], and airborne nano-pollution [55]. Climatic stresses involve factors such as cold [56,57], drought [23], flooding [12], ozone [15], heat [4], ultra-violet light exposure [58], and wind [59]. Biotic stresses include grazing [60], insects [61], bacterial [62], fungal [16], and viral pathogens [63]. These stresses may occur individually or in combination, such as heat and salinity [34,64]; waterlogging and drought [65]; heat and drought [30,66]; heat and pathogen stress [67]; heat and nutrient stress [68]; salinity, drought, and waterlogging stress [69]; or heat, drought, and ozone stress [70].

Heat stress can cause adverse impacts on plants during all growth stages, starting from germination through reproductive development and maturation. Under climate change, heat stress (when crops are subjected to temperatures > 35 °C) or extreme heat waves represent a serious threat to crops and their physiology [71], as well as on food, water, and energy security [72]. Temperature stress types can be classified into two main groups: heat stress (>35 °C) and cold stress, which may include chilling injury (15–0 °C) and freezing injury (<0 °C). Many changes in plant functional processes occur under higher temperatures, which negatively affect crop productivity. The reproductive stage is greatly hampered by heat stress in almost all plants [37]. This section explores examples of combined stresses that are associated with heat stress.

### 3.1. Heat and Drought Stress

**Figure 3:**
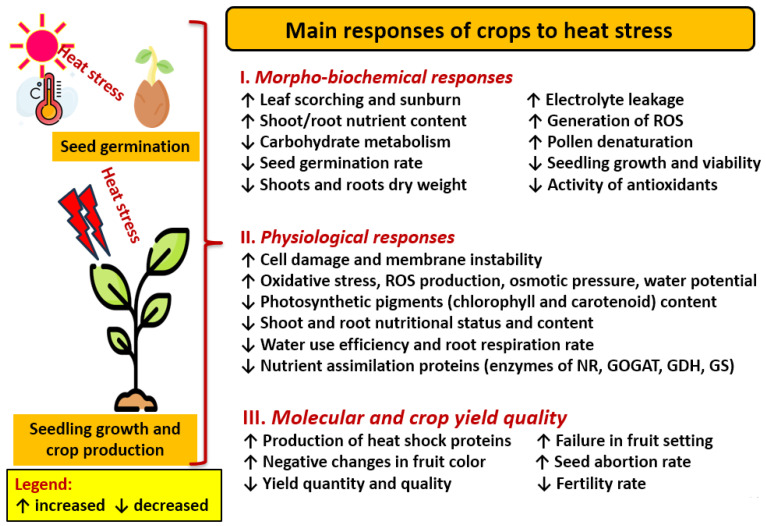
The main responses of crops to heat stress. The main attributes effected by these responses are morphological, biochemical, physiological, and molecular (Sources: [37,74,75,76,77]). The plant images were downloaded from https://www.flaticon.com/free-icon/plant_4147953 accessed on 3 March 2024. Abbreviations: reactive oxygen species (ROS), nitrate reductase (NR), glutamine oxoglutarate aminotransferase (GOGAT), glutamate dehydrogenase (GDH), glutamine synthetase (GS).

**Figure 4:**
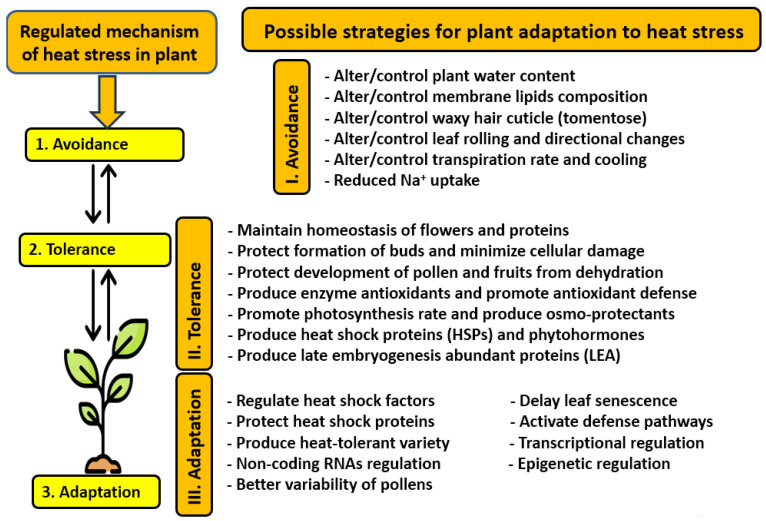
Possible strategies that crops can adopt against heat stress. These strategies include three broad options: avoiding the stress, tolerating the stress, and adapting to the stress. Sources: [9,37,74,75,77,88].

The main responses of cultivated plants to heat stress involves morphological, biochemical, physiological, and molecular attributes and processes (Figure 3). Heat stress can lead to damage at any plant growth stage, with a corresponding reduced crop yield. This requires urgent and timely management, as possible strategies to protect crops from heat stress include avoiding this stress as the first option. Subsequent strategies include how to create heat tolerance in plants and adaptations to address the heat stress (Figure 4).

Many studies have addressed strategies to deal with heat stress, e.g., [37,74,75,76,77]. Plants can evolve complex acclimation mechanisms to address stresses that can be tailored by stressed plants to be more suitable under stress combinations [78]. Therefore, plant responses to combined stresses cannot be easily predicted based on studies that investigated stresses individually [79]. Plant survival and growth under two or more stresses that occur simultaneously may be improved or reduced as compared to each stress individually, although most combined stresses have additive negative impacts [2]. Understanding the tolerance mechanisms of crops and how they interact under the variable environmental conditions driven by climate change is important to maintain the stability of crop yields under field conditions [2].

Due to the increasing mean global temperatures, combined heat and drought stresses are becoming more common and negatively affect the global farming system [2]. Combined drought and heat stress can cause significant reductions in crop yields, including tomato [80], wheat [81,82], maize [83], rice [84], soybean [85], chickpea [86], and potato [87]. Crop responses to combined heat and drought stress mainly depend on the presence of antistressors (i.e., phytohormones and amendments), along with the tolerance of such crops to these stresses (Table 1).

**Table 1:** Impacts of combined drought and heat stress on crops in the presence and absence of antistressors.

Plant Species	Stress Conditions (Heat and Drought)	Applied Antistressors (If Any)	Main Findings	Refs.
Tomato (*Solanum lycopersicum* L.)	Drought (40%FC), heat stress (38 or 45 °C (8 or 6 h)	Arbuscular mycorrhizal fungi (AMF)	AMF induce alterations in phytohormones and modulate aquaporin expression under stress	[80]
Soybean (*Glycine max* L.)	Heat (40 °C) and drought (30% reduction in pot weight) for 9 days	Jasmonic acid (JA) at 0, 50, 100 and 250 µM for 8 days	JA-priming protected seedling growth by improving the photosynthetic efficiency and potentiated antioxidant defense responses to alleviate oxidative stress	[85]
Potato (*Solanum tuberosum* L.)	Drought (PEG 8000; 10%) and heat (35 °C) for 15 days	Transgenic potato plants with no added antistressors	These plants minimized oxidative stress by activating antioxidants (Cu-Zn-SOD, Fe-SOD, CAT) and accumulation of osmo-protectants	[87]
Wheat (*Triticum aestivum* L.)	Drought (no watering of plants), heat (29 °C) for 5 days	No added antistressors, grown in two Biotron climate chambers	Negative impacts on biomass and yield, positive impact on most gluten parameters	[81]
Wheat (*Triticum aestivum* L.)	Heat (36 °C), drought (45–55% of soil water holding capacity)	No added antistressors, grown in artificial growth chambers	Combined stress reduced photosynthetic pigments and rate; the activities of SOD, POD, CAT, and GR. Plants increased osmo-regulation by forming soluble protein and sugar, proline	[89]
Cotton (*Gossypium hirsutum* L.)	Heat (37 °C), drought expressed as 25% water replacement	No added antistressors, grown for 4 weeks in growth chamber	Co-overexpression of RCA and AVP1 support transgenic cotton by increasing net photo-synthetic rate and seed fiber yield	[90]
Durum wheat (*Triticum turgidum* L.)	Heat (31–36 °C), drought (180 mm of irrigation water reduced from 500 mm for full irrigation)	No added antistressors, field study using drip irrigation	Studied glutenin fractions and the γ-gliadins were significantly reduced under stress, while β-gliadins were increased	[91]
Chickpea (*Cicer arietinum* L.)	Drought stress by withholding water for 3 days; heat at 32, 35 and 38 °C for 12, 6 and 2 h, resp.	No added antistressors, growth chamber; pots containing sand	Drought priming and consequent heat stress confirmed the role of heat shock proteins and heat shock factors and related tolerant gene families in studied plants	[86]
Wheat (*Triticum aestivum* L.)	Drought (50–55% field capacity for 8 days), heat stress (36 °C) for 3 days	No added antistressors, pots contained sand, soil, and farm yard manure	During vegetative growth drought priming activated plant defenses, antioxidative action; induced thermo-tolerance calmodulin, polyamine and glutathione synthesis genes	[92]
Winter wheat (*Triticum aestivum* L.)	Heat stress (38 °C), drought (water at permanent wilting point) for 14 days	No added antistressors, potted soils in a growth chamber	Stress reduces water availability on leaf gas-exchange parameters (photosynthesis, transpiration, and WUE) during stem extension stage inducing changes in grain yield	[93]

Abbreviations: polyethylene glycol (PEG), catalase activity (CAT), superoxide dismutase (SOD), peroxidase (POD), glutathione reductase (GR), Rubisco activase (RCA), Arabidopsis vacuolar H^+^-pyrophosphatase gene (AVP1), and water use efficiency (WUE).

Several reports on metabolic and physiological processes under combined heat and drought stress have been published. These reports focused on many issues, including the strong inhibiting role of both drought and heat stress in reducing the partitioning of carbon assimilates to the roots, increasing oxidative stress and the antioxidant capacity [2]. The role of signaling lipids was also reported, including sphingolipids, oxylipins, phosphatidic acid, phosphor-inositides, lyso-phospholipids, and N-acylethanolamines, for mediating the combined drought and heat stress [29]. The response of grass pea and its wild relatives to combined heat and drought stress was discussed by Aloui et al. [94], whereas the response of wild relatives of rapeseed mustard was reported by Kashyap et al. [95]. The complex plant response to heat and drought stress under climate change was reported by Puppala et al. [96] and Sato et al. [31].

### 3.2. Heat and Salinity Stress

Soil salinization has become common in many areas all over the world under increasing atmospheric temperatures, especially the arid and semi-arid regions [32]. Arid and semi-arid areas were estimated to be 33% of global irrigated arable soils suffering from salinization [97]. The main contributors to salinity stress (saline soil and irrigation water) may trigger ionic, oxidative, and osmotic stresses, which require the production of plant-protective compounds or enzymic antioxidants against ROS and osmo-protectant metabolites for regulation of the osmotic potential of plants [97]. High temperatures in arid zones can accelerate the formation of soil salinization, leading to more stress on crops [98]. It is expected that climate change will increase the salinization of soils under heat waves that vary in duration, intensity, and frequency [74,99,100,101]. Thus, the management of both heat and salinity stress in cropping systems will become increasingly important. This may involve agricultural practices like grafting [99], heat pre-treatment for seeds [102], seed nano-priming [103,104], or biotechnology approaches [105]. The application of antistressors has been used to mitigate combined heat and salinity stresses in crops (Table 2). The most promising nanomaterial antistressors used against combined heat and salinity stress in crops include nano-selenium [64], nano-silica [102], and silicon [106]. Phytohormones such as salicylic acid [33,107] and gibberellic acid [98] have also been reported as antistressors to support crop production under combined heat and salinity stress. The mechanisms of such combined stresses and its mitigation by plants still need more investigation.

### 3.3. Heat and Pathogenic Stress

**Figure 5:**
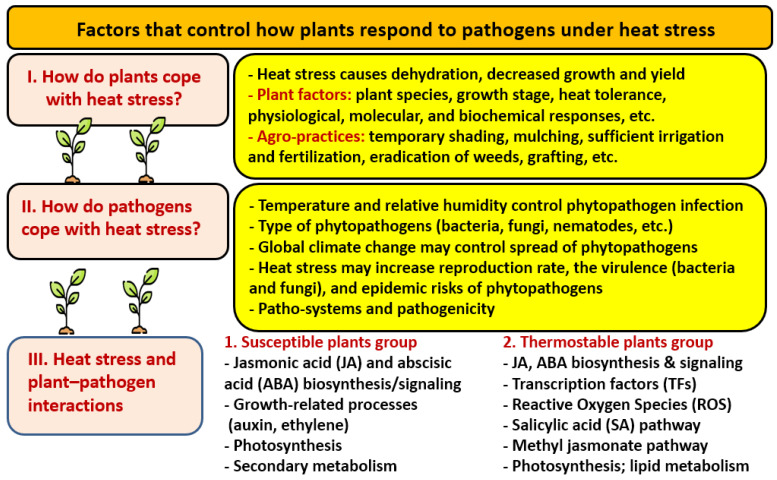
The mechanisms plants use to combat pathogens under heat stress. Adapted from [113].

Higher temperatures may support the growth and spread of phytopathogens. In a review on the effects of heat stress on plant–pathogen interactions, Desaint et al. [113] discussed the key features of combined heat and pathogenic stress (Figure 5). Under combined stress, the response of plants cannot be predicted from the evaluation of individual applied stresses [114]. Photosynthesis and carbon metabolism are expected to downregulate under individual and combined stresses through the activation of transcription factors (TFs). Under heat stress, plant susceptibility may increase due to the inhibition of plant defenses [113]. The negative effects of heat stress on plant resistances are not restricted to specific species of pathogens or plants and their related lifestyles. Generally, increased temperature negatively affects all types of resistance responses (i.e., pathogen-triggered immunity, effector-triggered immunity, and quantitative-disease resistance), although cases of immune response inhibition mainly concern effector-triggered immunity [113]. Studying the combined stress of heat and pathogens still needs more investigation that takes into account the complexity of the natural interactions among plants, a wide variety of pathogens (i.e., plant pathobiota), and the soil microbial community.

Many studies have reported negative impacts of heat and pathogens on crop production, such as grapes [115], wheat [116], chickpea [117], and peas [118]. On the other hand, the benefits of heat stress were evaluated as heat pre-treatment (priming) to support plant production under phytopathogen stress. This approach was confirmed for many plants, such as barley using brassinosteroids [119], soybean under nano-selenium application [120], *Arabidopsis* seedlings using *Bacillus cereus* for plant growth promotion [121], French bean (*Phaseolus vulgaris*) using serine peptidases [122], and barley (*Hordeum vulgare* L.) using heat shock [123]. Other reviews on this topic have addressed plant nutrient relations under heat stress [76], heat stress in a variety of crops [124,125,126,127], maize and heat stress [128], and rice breeding under heat stress [129].

## 4. Biogenic Nano-Agrochemicals

There are many groups of nano-agrochemicals, which can be classified into nanofertilizers, nano-biofertilizers, and nanopesticides, in which NPs are considered active ingredients in different forms, such as nano- and bioencapsulation, nanodelivery systems, etc. Nanopesticides may include nano-fungicides, nano-herbicides, nano-insecticides, nano-bactericides, and nano-nematicides. These nano-compounds also allow the targeted delivery of nutrients for crop productivity [130]. Nano-agrochemicals are considered sustainable alternatives to traditional agrochemicals, especially the biological and/or green forms of nano-agrochemicals [131,132]. The term biogenic nano-agrochemicals comes from using biological organisms (bacteria, fungi, algae, etc.); plant extracts (leaves, flowers, stems, etc.); or agro-industrial biowastes to produce nano-agrochemicals. The main function of these molecules is to act as natural capping, reducing, and stabilizing agents, which is critical to making NMs useful for agricultural purposes [133]. As compared to the physical and chemical approaches of making NMs, the biogenic techniques are economically viable, eco-friendly, and highly efficient. Physical and chemical synthesis of NMs have many problems. They are expensive, produce toxic byproducts, and require high energy input and axillary reagents [133]. Thus, the biological or green synthetic techniques to make NMs are considered a sustainable approach [134]. The process of preparing NMs using biogenic methods has been reported for many applications, such as biomedical applications [135]; nano-biofortification [136]; and using nanometals such as selenium [45], silver [137,138], iron [136], TiO_2_ [139], zinc/zinc oxide [140,141], and copper oxide [142] to address a variety of agronomic issues. The utilization of nanopesticides and nanofertilizers has been considered a solution for global food security and sustainable agricultural development [21,143]. Although nano-enabled agrochemicals can mitigate the toxicity of heavy metals [144], their ecological risks, fate, mobility, and ecotoxicology are still unknown, with the potential for significant threat to human health [145].

## 5. Nano-Management of Crop Production under Heat Stress

Due to climate change, plant growth and productivity face many adverse effects under higher temperatures, leading to heat stress [146]. Heat stress can alter all plant attributes, including the morphological, biochemical, and physiological processes in plants, leading to a significant reduction in plant growth and crop production [147]. This reduction is a result of the accumulation of reactive oxygen species (ROS), H_2_O_2_, and malondialdehyde (MDA). The most sensitive plant growth stage to heat stress is the reproductive stage, where heat stress causes a loss in flower buds and seed yield, along with chlorophyll degradation, photosystem II disruption, and inhibition of the photosynthetic process [146]. Management of heat stress in crops can be achieved though approaches such as mulching [148]; nutrient management [64]; the application of protectants including salicylic acid [149], trehalose [150], melatonin [35], and β-sitosterol and biochar [151]; molecular breeding strategies [4]; bacterial seed treatment [75]; and nano-management [64,146,152,153].

### 5.1. Biogenic Nanofertilizers under Heat Stress

The growing global population has led to an increase in food demand. Modern approaches emphasize increased fertilizer utilization for higher crop production. However, chemical fertilizers have low use efficiency and cause many environmental problems (Table 3). Nanofertilizers are a promising approach when they are applied at the right time in the correct dose and form. Some nanofertilizers show more promise than others, with biological nanofertilizers and slow-release nanofertilizers being particularly promising. In general, nanofertilizers (particle sizes 1–100 nm) have higher surface areas, higher fertilization efficiency, lower loss rates, higher microbial activity, a timely and balanced nutrient supply, and lower costs compared to traditional chemical fertilizers [154]. Problems associated with traditional chemical fertilizers include eutrophication, soil quality deterioration, groundwater and air pollution, and a diminished soil macro- and micronutrient supply capacity [155,156,157]. Biological nanofertilizers are preferable compared to other nanofertilizers due to lower cost, toxicity, and high safety levels, leading to sustainable farming [158].

**Table 3:** A comparison of nanofertilizers with traditional chemical fertilizers.

Comparison Item	Chemical Fertilizers	Nanofertilizers
Definition	All fertilizers that are synthetized from chemical materials in factories using industrial processes	Fertilizers at a nanoscale that can supply plants with nutrients, may include chemical or biological forms
Main methods of synthesis	Mainly from industrial chemical and physical methods	Physical (e.g., evaporation, laser ablation, and sputtering); chemical (e.g., vapor deposition, chemical reduction, and sol–gel synthesis); biological/green synthesis (microbes-mediated and biomimetic synthesis); recycling methods (e.g., mechanical attrition and electrochemical synthesis); and others
Main methods of application	Soil application, foliar spraying (phyllo-sphere), and seed priming	Soil application (rhizosphere), foliar spraying (phyllo-sphere), and seed priming
Types of fertilizers	Mainly depends on the type of nutrient in the synthetized fertilizer, such as N, P, K, or others. Often have high purity and supply only the nutrients they were designed to	Nanoscale additives (nanodelivery of nutrients as particles or emulsions), nanoscale fertilizers (controlled release of nano-nutrients by encapsulation), and nanoscale coatings (incorporated ingredients in the matrix of organic biochemical polymers, serving as carriers)
Types of composite fertilizers	There are single and combined fertilizers along with composite fertilizers like (19:19:19, N:P:K)	Hydroxyapatite, hydrogel, chitosan, graphene or carbon, zeolite, etc.
Factors that control nutrient-release, movement, translocation, and uptake by plants	Factors related to fertilizer, cultivated plants, soil properties and other environmental factors	Factors related to nano-nutrients (dose, size of NPs, surface, type, etc.); growing conditions (moisture, temperature, pH, salinity, etc.); plant species (roots, growing stage, etc.); and growth media (rhizosphere, pH, and microbial activity)
Advantages of such fertilizers	High solubility and high uptake rate by cultivated plants, relatively inexpensive supply of nutrients	Higher nutrient use efficiency, timely and balanced nutrient supply, improved edaphic features, higher microbial activity and soil amelioration, lower loss rate, production cost, vol/wt. other than the traditional due to their tiny size and larger surface area, promote precision farming, water holding capacity
Main proposed problems	Soil quality deterioration, eutrophication, groundwater pollution, and air pollution	Nanotoxicity to plants, microbes, food chain, water, air, and human health
Main loss pathways	Loss by leaching, evaporation, and surface runoff	Loss by evaporation, drifting, surface runoff, hydrolysis, and photolytic degradation of nutrients

Sources: [73,111,154,155,156,157].

Many studies have focused on biogenic nanofertilizers and discussed nutrients such as CuO- and ZnO-NPs [159], Zn-NPs/ZnO-NPs [140,160], Si-NPs [161], phosphorous NPs [162], silver and iron NPs [163], MgO-NPs [164], and CuO-NPs [165]. Many of these reports have focused on sustainable agriculture [132,158,166], but few have addressed heat stress (Table 4). The impact of these nanofertilizers on heat stress depends on the synthesis process, size of the NPs, and plant species, which control the plant tolerance to heat stress through the action of osmo-protectants, phytohormones, and signal molecules [167]. These plant-produced molecules are more bioavailable and effective after applying NMs or NPs, as they facilitate the delivery of plant-produced molecules to their action sites [168]. Foliar application of many nano-based nutrients (e.g., SiO_2_-NPs, Se-NPs, ZnO-NPs, and CuO-NPs) has been reported to be effective against heat stress (Table 4). These NPs can enhance plant growth and performance by supporting the plant defense system through increased antioxidant enzyme activity, accumulation of proline, gas exchange, photosynthetic apparatus efficiency, etc.

The mechanisms underlying the NP-mediated regulation of stress responses may differ from one NP to another; there is a need to understand the transcriptomics in general [167], along with the plant-associated microbiomes and engineering [17]. For example, CeO_2_-NPs as nano-enzymes reduced heat damage to photosystems by scavenging ROS in plant chloroplasts and increased the quantum yield in photosystem II, carbon assimilation rate, and rubisco carboxylation by 19, 67, and 61%, respectively, compared to the control [176]. There is a need for more research on biogenic nanofertilization that investigates different nanofertilizer and crop combinations.

Treating plants under heat stress with NPs can reduce such stress by triggering one or more molecular reactions through the production of enzymes and proteins, transcription factors, and gene controls that support plant survival [22]. For example, Si applied at 1.66 mM on wheat seedlings under heat stress (45 °C, 4 h) stimulated the overexpression of genes for the aquaporins TaPIP1 (*Triticum aestivum* plasma membrane intrinsic protein) and TaNIP2 (*Triticum aestivum* nodulin 26-like intrinsic protein) in wheat but not Si-NPs [177]. On the other hand, the study of the molecular impacts of ZnO-NPs on *Arabidopsis thaliana* seedlings during heat stress (37 °C) showed TGS (transcriptional gene silencing) in aerial leafy tissues and the improved alleviation of TGS-GUS (-glucuronidase) genes [172]. The most significant impact of applied NPs was the upregulation of genes encoding for the large and small subunits of RUBISCO, chlorophyll a/b-binding proteins, and phosphoenolpyruvate carboxylase (PEPC), which, in turn, caused an increase in photosynthesis, altered the energy metabolism, and decreased the H_2_O_2_ concentration [178,179].

### 5.2. Biogenic Nanopesticides for Crop Protection

**Figure 6:**
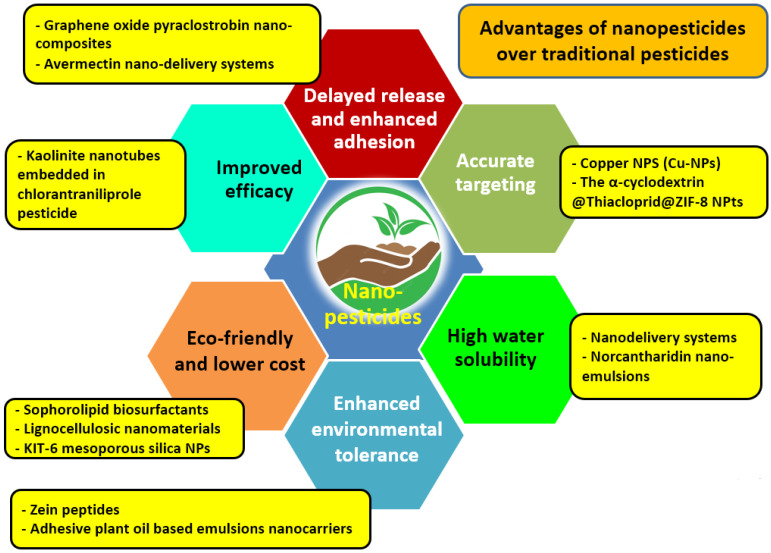
Advantages of nanopesticides (NPts) as compared to traditional pesticides. Examples of NPts are presented in the yellow boxes. Source: [182].

There are many kinds of pesticides, depending on their nature (plant-derived or mineral oil-based) or application (e.g., insecticides, herbicides, fungicides, algicides, nematicides, bactericides, rodenticides, etc.). Traditional pesticides may cause several environmental problems due to runoff and chemical processes like hydrolysis, biodegradation, bioaccumulation, and photodegradation [180]. Thus, the extensive use of traditional pesticides has led to adverse impacts on the agroecosystem and human health [181]. Nanopesticides can be used for crop disease and pest management and come in different formulations, such as nanocarrier-based pesticides and nano-emulsion-based surfactants incorporated with metal or metal oxide-NPs as active ingredients (AI) [182]. Nanopesticides represent a promising solution with exceptional performance advantages over conventional pesticides [130]. These benefits include reduced environmental pollution risks; improved chemical stability, efficiency, and target delivery; and good biocompatibility as compared to conventional pesticides [180] (Figure 6).

To avoid the problems of traditional pesticides, alternatives are needed, like bio-based pesticides that are derived from organic sources, including microbes, animals, plants, and fungi. These bio-based pesticides are safer and more eco-friendly than traditional chemical pesticides. Along with bio-pesticides, nanopesticides are important alternatives that include metal/metalloid-based nanopesticides (e.g., Ag, Se, CuO, TiO_2_, SiO_2_, and ZnO) and nanocarrier-based pesticides composed of many materials, such as lipids, polymers, and proteins [130]. Other forms include nano-silica pesticides [183], nano-emulsion-based pesticides [184], polymer-based nanopesticides [185], chitosan-based nanopesticides [186], and nano-capsule-based pesticides [187]. There are metallic oxide-NPs that have fungicidal and antimicrobial properties that make them useful against many phytopathogens. These include nano-TiO_2_, nano-ZnO, and nano-Fe_2_O_3_ [188,189]. Many nanomaterials have been used to control phytopathogens (bacterial, fungal, and viral; Table 5), along with the biogenic approach for sustainable agriculture [133].

Many different formulations of nanopesticides have been produced through biogenic or chemical approaches using metals/metalloids such as Ag, SiO_2_, CuO, and ZnO, along with nanocomposites like SiO_2_/Ag nanocomposite or chitosan [187,190,191,192]. As far as we know, there are no studies on the direct impact of applied geogenic nanopesticides under heat stress on cultivated plants. Investigations have included studies on the thermo-responsiveness of nanopesticides [193,194]; controlled release by nanopesticides [195]; and factors that control the release of active ingredients from nanopesticides under heat stress, along with other factors, including pH, light, redox status, enzymes [129], and the release of nanopesticides at high temperatures [196]. High temperatures may cause an increase in the level of several phytopathogens and microbial disease outbreaks, particularly under higher humidity, because temperature directly controls the reproduction and metabolism of microbes [197]. Temperature not only controls phytopathogen invasion but also potentially the extent of plant host damage during infection through its impact on plant pathogen enzymatic activities and toxin production [198]. Thus, the study of biogenic nanopesticide performance in crops under heat stress still needs more investigation.

**Table 5:** Impacts of nanopesticides on crop production and their anti-pest activity.

Plant Species or Culture Used	Nano-Based Pesticide (Applied Dose and Size)	Pathogen Studied	Main Effect	Refs.
Common Bean (*Phaseolus vulgaris* L.)	Nano-Se + SiO_2_, 50 ppm of each	Fungal pathogen (*Alternaria alternata* L.)	Effective alternatives to traditional fungicide to control A. alternata in common bean	[199]
Pine (*Pinus thunbergii* Parl.)	Nano-Cu-BTC (10 ppm)	Pine wilt nematode (*Bursaphelenchus xylophilus*)	Effectively controlled Japanese pine sawyer vector insect by delivering avermectin absorbed by the insect larvae	[200]
Lentil (*Lens culinaris* medik.)	ZnO-NPs (100 ppm) (<100 nm)	Bacterial pathogen (*Xanthomonas axonopodis* pv. phaseoli)	Reduced blight, wilt, nematode multiplication, and leaf spot disease severity indices and increase plant growth	[201]
Rice (*Oryza sativa* L.)	ZnO-NPs (4, 8, and 16 ppm) (48.2 nm)	Bacterial pathogen (*Xanthomonas oryzae* pv. oryzae)	Effective anti-microbial agents against bacterial leaf blight of rice	[202]
In vitro assay (nutrient agar medium)	Biogenic Ag-NPs (100 ppm) (55 nm)	Bacterial pathogen (*Ralstonia solanacearum*)	Inhibited the bacterial activities by damaging the pathogen’s cell membrane	[203]
In vitro assay (nutrient agar medium)	Green Ag-NPs (10 ppm) (from 24.5 to 43.1 nm)	Bacterial pathogen (*Ralstonia solanacearum*)	An effective eco-friendly anti-bacterial agent inhibited R. solanacearum up to 80%	[204]
In vitro assay (nutrient broth media)	Green Ag-NPs (78 to 500 ppm) (from 23 to 63 nm)	Bacterial pathogen (*Ralstonia solanacearum*)	Effective alternative bacterial agent to control tomato wilt	[205]
In vitro assay (nutrient agar medium)	Biogenic SiO_2_/Ag nanocomposite	Bacterial pathogen (*Ralstonia solanacearum*)	Effective and eco-friendly antibacterial agent, embedded in mesoporous nano-SiO_2,_ avoids aggregated Ag-NPs	[206]
In vitro assay (nutrient agar medium)	Biogenic Ag-NPs (100 ppm) (55 nm)	Fungal pathogen (*Fusarium oxysporum*)	Mycelial growth of fungi was reduced up to 40–50%	[203]
In vitro assay (poisoned food technique)	ZnO-NPs (10, 100, and 1000 ppm) (<50 nm)	Fungal pathogen (*Alternaria alternata* L.)	Nano-fungicide had higher efficacy than bulk form (ZnSO_4_)	[207]
In vitro assay (using broth culture)	Green ZnO-NPs (25, 50, 100, and 140 ppm) (30–40 nm)	Fungal pathogen (*Fusarium graminearum* L.)	Reducing deoxynivalenol and zearalenone controlled growth and mycotoxins	[208]
In vitro assay (poison food technique)	ZnO-NPs (100 and 800 ppm) (20–60 nm)	Fungal pathogen (*Fusarium moniliforme*)	Antimycotic potential of NPs inhibited hyphal growth, depending on particle size	[209]
Tomato (*Lycopersicon esculentum* L.)	Silica-NPs (100, 200, 300 and 400 ppm) (10.7 nm)	Fungal pathogen (*Alternaria solani*)	Reduced disease severity as an eco-friendly and safe alternative to chemical fungicides	[210]
Table grape (*Vitis vinifera* L.)	Chitosan silica nanocomposites	Fungal pathogen (*Botrytis cinerea*)	Reduced fungal growth 100% by inducing enzymatic activity and gene expression levels	[211]
Tobacco (*Nicotiana benthamiana* L.)	ZnO-NPs (100 ppm), (18 nm)	Viral pathogen (tobacco mosaic virus)	Marked suppression of viral invasion in the inoculated leaves by increasing SA and ABA phytohormone levels	[212]

Abbreviations: Polymers of copper and trimesic acid (Cu-BTC), salicylic acid (SA), and abscisic acid (ABA).

## 6. Potential and Limitations

As mentioned before, the biogenic approach to produce nano-agrochemicals has several benefits compared to chemical and physical approaches. This includes the low toxicity and risk to the agro-environment, along with the low cost of biogenic products. Many ecological risks do exist under the excessive application of nano-agrochemicals, with the level of risk primarily depending on the size, shape, and biodegradability of the NMs [6]. Several studies have addressed this concern, including Chaud et al. [213], Grillo et al. [214], Maity et al. [215], Mubeen et al. [21], and Victoria et al. [180]. Ecotoxicological risks can be reduced by applying nano-agrochemicals at the right time in the correct dose to suitable crops. Environmental conditions (e.g., temperature, light, humidity, and the microbial community) control the biodegradation of such nanomaterials, their persistence, and their toxicity risks. Several questions need to be addressed as regards nanomaterial use in the agricultural setting:

- Are phytopathogens able to sense plant heat stress, and, if so, are they involved in virulence?
- How does heat stress affect microbial virulence at the metabolic and transcriptional levels?
- How can we engineer plants to be resistant to heat stress using biogenic nano-agrochemicals?
- How do plants adapt to heat stress, and how can biogenic nano-agrochemicals support this adaptation?
- To what extent can we produce biogenic nano-agrochemicals at the industrial scale?
- To what extent can we apply biogenic nano-agrochemicals against heat stress in the greenhouse and at the field level?
- What is the biocompatibility of biogenic nano-agrochemicals?
- What are the key functional groups in nanomaterials for assessing their nanotoxicity?
- Do biogenic nano-agrochemicals have a negative impact on non-target organisms under heat stress?
- What are the expected nanotoxicity and risks of biogenic agrochemicals over the long term in agro-ecosystems in the presence and absence of heat stress?
- To what extent is a multidisciplinary approach needed to evaluate the impact of biogenic nano-agrochemicals?

## 7. Conclusions

Global climate change is a threat to food production. Higher temperatures are a common feature of climate change. Heat stress threatens food security as an individual stress and in association with other stresses like drought, salinity, and pathogenic stress. This leads to many problems for crop production. The use of nano-agrochemicals is a promising approach to mitigate heat stress and associated stresses, with a focus on nanofertilizers and nanopesticides. Compared to traditional chemical approaches, the biogenic nano-strategy is an emerging potential solution for sustainable agricultural production. These biogenic nano-agrochemicals are preferable due to their cost-effectiveness, high bioavailability, biocompatibility, and high potential to synthesize their NMs. Although biogenic nano-agrochemicals are superior alternatives to traditional agrochemicals for the control of phytopathogens and to supply nutrients, the ecological safety aspects of NM use in agriculture still need more investigation.

## Figures and Tables

**Table 2:** Impacts of combined salinity and heat stress on crops in the presence and absence of antistressors.

Plant Species	Stress Conditions (Heat and Salinity)	Applied Antistressors (ASs)	Main Findings	Refs.
Cucumber (*Cucumis sativus* L.)	Heat stress (41 °C); salinity stress (EC 4.49 dS m^−1^)	Nano-Se (25 mg L^−1^), silicon (Si, 200 mg L^−1^)	Applied nano-Se and Si promoted plant growth and yield under stress by controlling stomatal opening and regulating the osmotic balance	[64]
Soybean (*Glycine max* L.)	Heat pre-treatment of seeds (45 °C), salt stress (sea water (diluted by 1/12 and 1/6)	SiO_2_-NPs (1 mM; 50 nm) and nano-Se (20 ppm; 40 nm)	Heat pre-treated seeds in the presence of nano-antistressors ameliorated salt-stress and recovery against oxidative stress	[102]
Oregano (*Origanum vulgare* L.)	Heat (27 °C), salt stress (50, 75, 100, 150, 175 mM NaCl)	SA (1 mM), GABA (0.5, 0.7, 1.0, 1.5 and 2.0 mM)	Applied SA and GABA compounds protected plants under stress by regulation of secondary metabolites and enzyme-pigments	[33]
Cherry tomato (*Solanum lycopersicum* L.)	Salt stress (100 mM NaCl) and heat (42 °C; 4 h/day)	Without-ASs (seedlings were grown in pots for 21 days in a growth chamber)	Combined stresses negatively impacted growth and photosynthetic pigments more than individual stresses, transcript accumulation and protein content depleted in stressed plants, reduced carbon assimilation	[108]
Rice (*Oryza sativa* L.)	Heat (31 °C) and salinity stress (75 mM NaCl)	Without-ASs (seedlings were studied under seedling, vegetative and reproductive stage)	Stress greatly reduced plant growth performance and yield. Heat stress did not impact yield during reproduction but reduced grain quality	[34]
Peppermint (*Mentha piperita* L.)	Salt stress at 60 and 120 mM NaCl and heat stress at 35 °C	Without-ASs (seedlings were grown in pots filled with soil, sand, manure and vermicompost in a ratio of 1:1:1:2, resp.)	Rosmarinic acid, soluble sugar, chlorophyll and K^+^/N^+^ decreased by 3.2, 1.8, 4.6 and 9 times after 72 h respectively at 35 °C and salt stress of 120 mM	[109]
White goosefoot (*Chenopodium album* L.)	Salt stress (100 and 300 mM diluted from sea water), heat (shaded and non-shaded plots)	Without-ASs (seedlings were grown under field conditions for 60 days)	Combined stresses had negative impacts on studied plants, shading improved plant tolerance to salinity and alleviated heat and drought stresses	[110]
Rice (*Oryza sativa* L.)	Soil salinity was 20 dS m^−1^), heat stress (35 °C/26 °C for day/night cycle)	Without-ASs (14 day old seedlings were grown in a growth chamber in pots)	Combined stress formed CAT, APX, SOD with rapid readjustment at the molecular and physiological levels	[111]
Rice (*Oryza sativa* L.)	Heat (30 °C), salt stress (75 mM NaCl)	Without-ASs (seedlings were grown in a hydroponic system for 14 days)	Under combined stress, specific genes can show molecular response along with physiological and metabolic mechanisms	[112]

Abbreviations: salicylic acid (SA), gamma-aminobutyric acid (GABA), catalase activity (CAT), superoxide dismutase (SOD), and ascorbate peroxidase (APX).

**Table 4:** Impacts of applied nanofertilizers or nanomaterials on plants under heat stress.

Plant Species	Nano-Based Nutrient Dose(s)	Nanofertilizer Synthesis (Size)	Heat-Stress Details	Main Effects	Refs.
Alfalfa (*Medicago sativa* L.)	ZnO-NPs (30, 60, 90 ppm)	Chemical (10–20 nm)	45 and 34 °C day/nighttime for 7 d	Pre-treatment seedlings alleviated heat stress by reducing ultrastructural damages (chloroplast, mitochondria, and cell wall)	[152]
Mung bean (*Vigna radiata* L.)	ZnO-NPs (15, 30, 45, and 60 ppm)	Chemical (20 nm)	40/25 °C day/night for 3 months	Up-streamed production of osmolytes and antioxidants to attenuate the shocks of heat stress	[146]
*Chrysanthemum morifolium* Ramat	Nano-Se (50, 100, 150 and 200 ppm)	Biological (50–200 nm)	37.3 to 41.6 °C for 3 months	Improved the antioxidant system, floral quality, and attributes	[169]
Wheat (*Triticum aestivum* L.)	Nano-Se (50, 75, and 100 ppm)	Biological (56–88 nm)	Heat stress (temperature not reported)	Reduced the incidence of wheat crown and root rot diseases, enhanced plant growth, and grain quality	[25]
Wheat (*Triticum aestivum* L.)	Ag-NPs (25, 50, 75 and 100 ppm	Green (34 nm)	35–40 °C for 3 h/day for about 3 days	Protected plants against heat stress through ROS and antioxidant defense	[170]
Sorghum (*Sorghum bicolor* L. Moench)	Se-NPs (100, 250, and 500 ppm) for 48 h	Chemical (10–40 nm)	38/28 °C (day/night) for 10 days	Improved germination of pollen, antioxidant activity, and increased seed yield	[171]
*Arabidopsis thaliana* L.	ZnO-NPs (0.1, 0.5 and 1 ppm)	Chemical (20 nm)	37 °C for 3 h	Enhanced the alleviation of heat stress by inducing transcriptional gene silencing/β-glucuronidase	[172]
Wheat (*Triticum aestivum* L.)	ZnO-NPs (1.5 and 10 ppm)	Chemical (25 nm)	32 °C for 12 days	Improved antioxidants, membrane stability, and reduced MDA and H_2_O_2_ content at 1.5 ppm	[173]
Wheat (*Triticum aestivum* L.)	Nano-sized chitosan-glycine-betaine (100 mM) for 18 h	Nanocomposite	37/28 ± 2 °C (day/night) for 14 days	Increased the activities of antioxidants, which aided in scavenging the stress-induced oxidative damages	[153]
*Arabidopsis thaliana* L.	Cerium oxide NPs (nanoceria)	Poly(acrylic acid) nanoceria (10.3 nm), multiple ways to synthesize	35 °C for 2.5 h	Reduced ROS, oxidative damage, and improved photosynthesis and carbon assimilation	[174]
Bell pepper (*Capsicum annum* L.)	Nano-encapsules of amino levulinic acid	Nano-encapsulation (78 to 94 nm)	at 35 °C for 6 h	Increased antioxidant enzymes (POD, SOD, and CAT) and proline levels under heat stress conditions	[175]

Abbreviations: malonaldehyde (MDA), reactive oxygen species (ROS), peroxidase (POD), superoxide dismutase (SOD), and catalase (CAT).

## Data Availability

No new data were created in this study.
